# Efficient Direct Cytosolic Protein Delivery via Protein-Linker Co-engineering

**DOI:** 10.1021/acsami.5c02360

**Published:** 2025-04-30

**Authors:** Lixia Wei, Heyun Wang, Melis Özkan, Andrada-Ioana Damian-Buda, Colleen N. Loynachan, Suiyang Liao, Francesco Stellacci

**Affiliations:** § Institute of Materials Science and Engineering, École polytechnique fédérale de Lausanne, Lausanne 1015, Switzerland; † Institute of Bioengineering, École polytechnique fédérale de Lausanne, Lausanne 1015, Switzerland; ‡ Institute of Biomaterials, Department Materials Science and Engineering, Friedrich-Alexander-Universität, Erlangen 91054, Germany

**Keywords:** protein therapeutics, cytosolic delivery, membrane permeability, arginine-mimicking ligand, cell penetration, protein functionalization

## Abstract

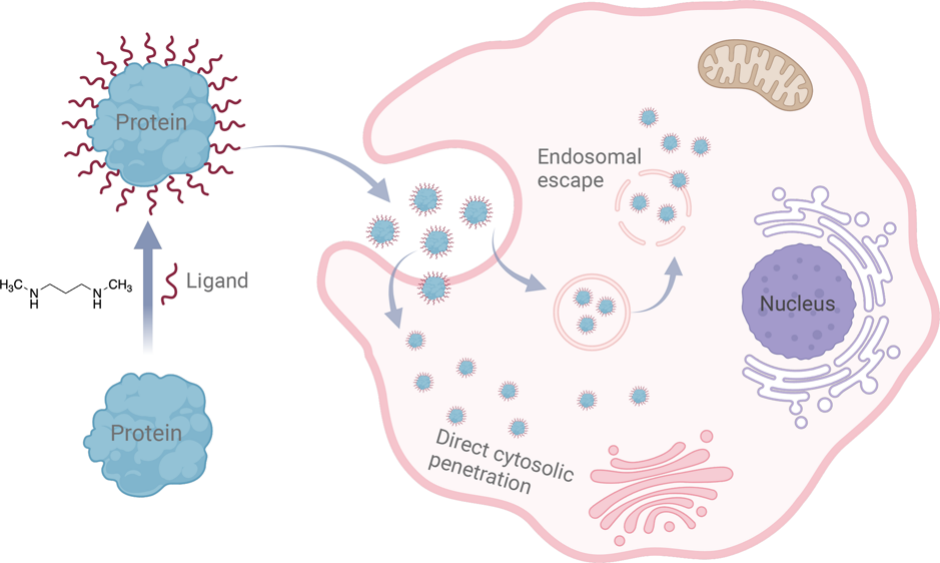

Protein therapeutics have enormous potential for transforming the treatment of intracellular cell disorders, such as genetic disorders and cancers. Due to proteins’ cell-membrane impermeability, protein-based drugs against intracellular targets require efficient cytosolic delivery strategies; however, none of the current approaches are optimal. Here, we present a simple approach to render proteins membrane-permeable. We use arginine-mimicking ligand *N*,*N*′-dimethyl-1,3-propanediamine (DMPA) to functionalize the surface of a few representative proteins, varying in isoelectric point and molecular weight. We show that when these proteins have a sufficient number of these ligands on their surface, they acquire the property of penetrating the cell cytosol. Uptake experiments at 37 and 4 °C indicate that one of the penetration pathways is energy independent, with no evidence of pore formation, with inhibition assays indicating the presence of other uptake pathways. Functional tests demonstrate that the modified proteins maintain their main cellular function; specifically, modified ovalbumin (OVA) leads to enhanced antigen presentation and modified cytochrome *C* (Cyto *C*) leads to enhanced cell apoptosis. We modified bovine serum albumin (BSA) with ligands featuring different hydrophobicity and end group charges and showed that, to confer cytosolic penetration, the ligands must be cationic and that some hydrophobic content improves the penetration efficiency. This study provides a simple strategy for efficiently delivering proteins directly to the cell cytosol and offers important insights into the design and development of arginine-rich cell-penetrating peptide mimetic small molecules for protein transduction.

## Introduction

Proteins are essential and are involved in almost every cellular process. The main advantages of protein-based therapeutics are their high specificity and potency. Besides, they are also accompanied by good biocompatibility and low toxicity. The number of protein-based therapeutics is exponentially increasing. Five of eight (excluding two vaccines due to special pandemic reasons) top-selling drugs globally in 2023 are protein drugs.^1^ However, due to the intrinsic physicochemical properties (primarily high molecular weight and surface charge), most current protein therapeutics are limited only to being used in the extracellular environment, for example, antibodies. Nevertheless, there is enormous potential in the development of intracellular protein therapeutics to treat cell disorders such as genetic disorders and cancers, for instance, gene editing enzymes, protein antigens, and CRISPR/Cas-based therapeutics.^2,3^ Yet, the development of these protein-based drugs against intracellular targets is hampered by the lack of efficient cytosolic delivery strategies and the susceptibility of proteins to enzymatic degradation.

To overcome this problem, a common strategy is to use transfection to deliver a plasmid encoding the protein of interest to the target cell^4,5^; in this case, the amount of protein supplied and the frequency of administration cannot be controlled. Other strategies, such as electroporation,^6^ microinjection,^7^ or using protein carrier lipid nanoparticles,^8−11^ have also been widely used. Besides these physical, mechanical, and biological methods, chemical modification of proteins, for example, complexation with fluoroamphiphiles,^12^ coordination with dendrimers,^13,14^ or use of cell-penetrating peptides (CPPs),^3,15^ has also been investigated. These methodologies show great promise in intracellular protein delivery, but challenges lie in the variation of proteins’ physicochemical properties, such as molecular weights, folding structures, surface charge distribution, hydrophobicity, and isoelectric points (pI). In most cases, electrostatic interaction plays a critical role in the binding of cargo proteins to their carrier.^16^ To resolve this issue, proteins have been either modified with specific tags to strengthen the binding between cargo proteins and carriers or directly conjugated onto the carriers via dynamic covalent linkages.^17−25^ These strategies are usually associated with extra synthetic and purification processes. Besides, there is the possibility that some chemical and biological modifications on cargo proteins may alter the protein activity. Another common issue with some existing carriers is the entrapment of cargo proteins in acidic compartments after endocytosis.^26,27^ This limitation will lead to a much decreased protein activity after intracellular delivery. To resolve this, researchers developed endosomolytic peptides, lipids, or surfactants to assist cargo proteins escaping from endolysosomes.^20,24,25,28,29^

Cationic nanoparticles and cell-penetrating peptides have been designed to enter cells in an energy-independent fashion, escaping the traditional endocytosis route, which is known as direct translocation.^30−32^ Recent studies have shown that glutamate (E)-tagged proteins with arginine (R) functionalized nanoparticles cooperate well to deliver a range of proteins directly into the cytosol in vitro.^33,34^ Although the molecular mechanisms of how arginine-rich peptides or nanoparticles enter cells have not been fully understood and remain debated, they indeed have attracted a lot of attention as one of the most promising carriers for intracellular delivery of therapeutic molecules. Analysis of some common CPP sequences, they always contain several basic amino acids, including arginine and lysine, which are positively charged, or they are hydrophobic CPPs.^35−39^

**Scheme 1:**
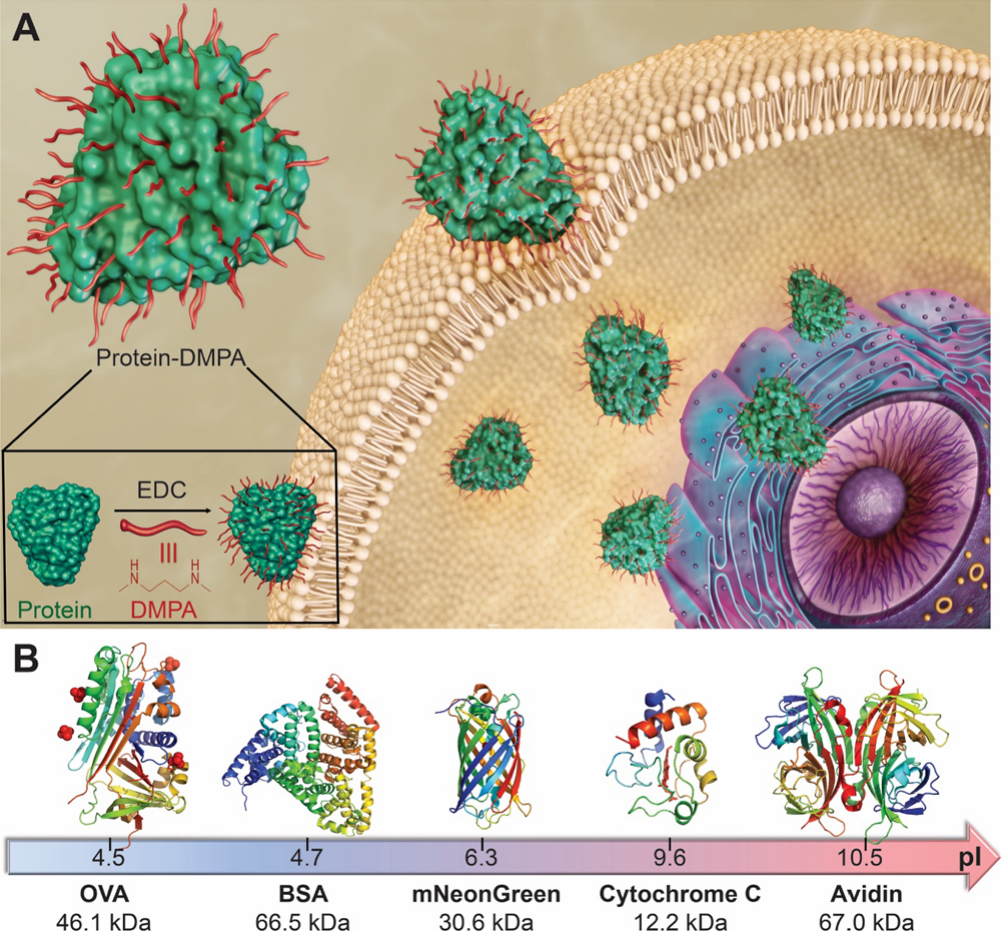
Schematic Illustration of Direct Cytosolic Delivery of Various Proteins through Simple Chemical Conjugation of DMPA Ligands (A) Illustration of chemical conjugating ligand DMPA on the surface of protein and modified proteins translocate the cell cytoplasm directly; (B) illustration of five investigated proteins, including their structures, isoelectric points, and MWs.

Inspired by these studies, we developed a protein cytosolic delivery strategy based on chemical conjugating of short arginine-mimicking cationic ligands. The ligand that we used features two secondary amine terminal groups, providing positive charges, and three alkyl groups, providing hydrophobicity. Through a one-step chemical conjugation via 1-ethyl-3-(3-dimethylaminopropyl) carbodiimide (EDC) coupling in aqueous solution, we can successfully conjugate DMPA onto the carboxyl groups of proteins without cross-linking them (Scheme 1A). To demonstrate the feasibility of this concept, here we used five different proteins, Ovalbumin (OVA), Bovine serum albumin (BSA), mNeonGreen, Cytochrome *C* (Cyto *C*), and Avidin (Scheme 1B), by varying their isoelectric points (pI) from acidic 4.5 to basic 10.5, their molecular weight (MW) from 12 to 67 kDa, and their different protein structures. Through applying one-step EDC coupling reaction, we show that all these proteins bypass classical endocytic pathways to directly translocate cargo protein into the cytoplasm by a combination mechanism of energy-independent direct translocation and lipid raft-dependent membrane fusion. A systematic study on the ligands further shows that the ligands must be cationic and contain hydrophobic aliphatic chains to confer cell penetration. These promising outcomes offer us an alternative solution for efficient direct cytosolic delivery of protein therapeutics.

## Results and Discussion

### Preparation and Characterization of DMPA Conjugated Proteins

The chemical reaction between protein and ligand DMPA relies on surface accessible carboxyl groups (−COOH) on the protein and secondary amines (−NH−) on the DMPA ligand. Here we used a commonly used coupling reagent, EDC, for activating −COOH groups and then reacting with −NH– groups on the ligand DMPA in 4-morpholinoethanesulfonic acid (MES) buffer, pH 4.7. In this reaction, the mass ratio of protein, ligand, and EDC is fixed into protein:ligand:EDC = 1:20:10, and we kept the protein reaction concentration ≤1 mg/mL to avoid cross-linking of the proteins with the diamine-terminated ligands. This one-step chemical synthesis approach is displayed in Scheme 1A.

The modified proteins were carefully characterized using a range of techniques to evaluate the size by dynamic light scattering (DLS), the surface charge by surface zeta potential, and the ligand density by matrix-assisted laser desorption/ionization time-of-flight (MALDI-TOF) mass spectrometry (Table 1).

**Table 1:** Physicochemical Properties of Native and Ligand-Modified Proteins

entry	sample/characterization	isoelectric point (pl)	DLS (nm)	zeta potential (mV)	mass spectrum (MW)	number of ligand/protein	basic group surface/total
1	OVA	4.5	4.2 ± 0.8	–8.3 ± 0.8	44,460		30/35
2	OVA-DMPA		4.6 ± 0.8	21.1 ± 4.7	45,380	9	39/44
3	BSA	4.7	6.1 ± 1.5	–7.3 ± 3.8	66,346		67/86
4	BSA-DMPA		8.2 ± 2.0	31.2 ± 4.5	72,057	56	123/142
5	mNeonGreen	6.3	4.5 ± 0.8	–1.2 ± 6.2	30,464		17/33
6	mNeonGreen-DMPA		5.0 ± 1.0	23.6 ± 4.5	31,763	13	30/46
7	Cyto C	9.6	1.2 ± 0.3	2.0 ± 5.6	12,179		20/21
8	Cyto C-DMPA		2.2 ± 0.3	18.2 ± 12.9	13,084	9	29/30
9	Avidin	10.5	6.3 ± 1.3	5.6 ± 2.3	63,564		49/68
10	Avidin-DMPA		8.6 ± 1.4	20.4 ± 47.2	66,098	25	74/93

**Figure 1:**
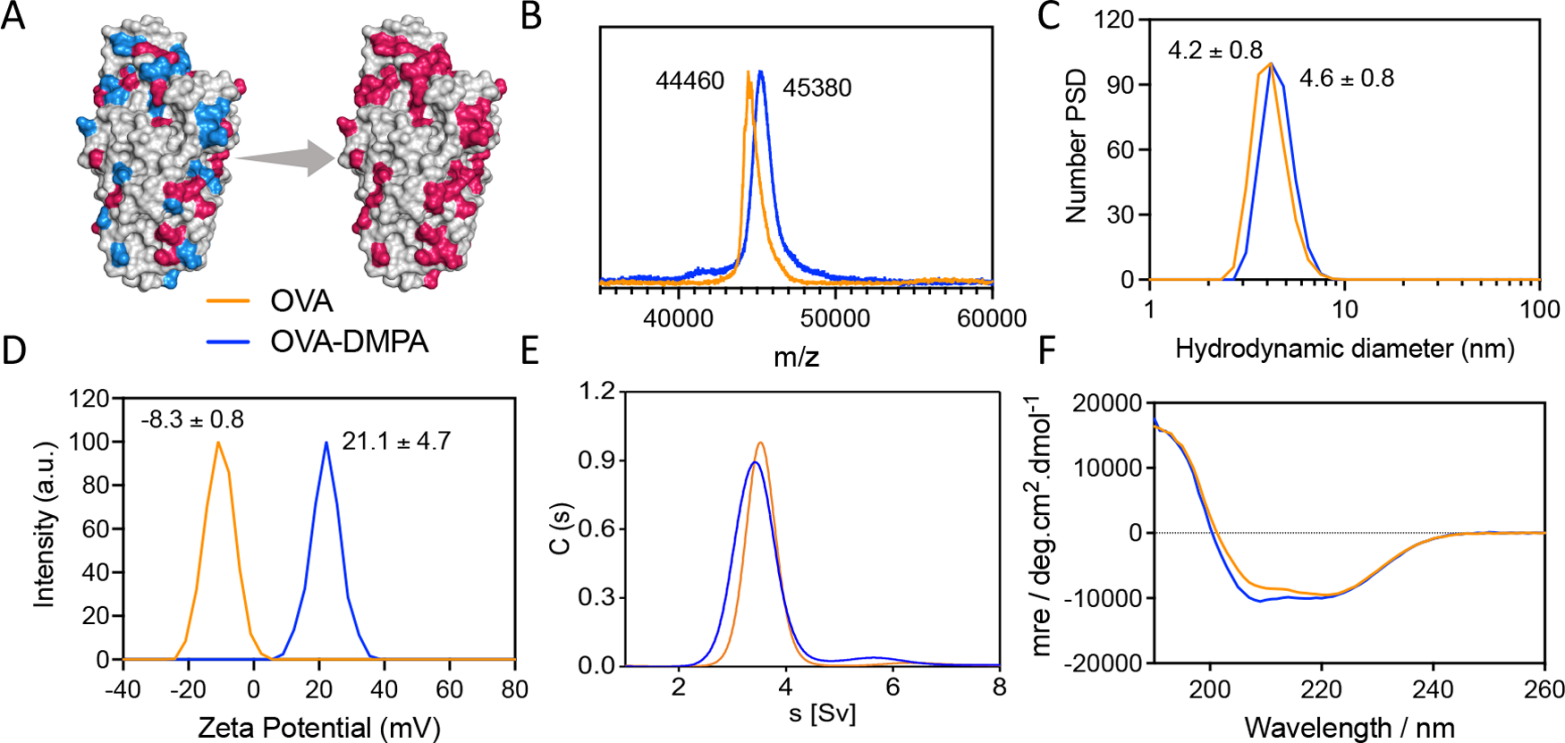
Characterizations of the ligand DMPA modified protein OVA. (A) Schematic illustration of surface modification of OVA protein by conjugating ligand DMPA onto the surface accessible negatively charged carboxyl groups (blue) of the protein, making it more positively charged (red). (B) MALDI-TOF mass spectrometric analysis of native and ligand modified OVA protein. (C) Size and size distribution measurement of OVA and OVA-DMPA by DLS. (D) Surface zeta potential of native and modified OVA protein. (E) AUC analysis of OVA and OVA-DMPA’s sedimentation behavior. (F) CD spectra of native and modified OVA’s secondary structure.

To prove the concept, we first used OVA as a model protein core and demonstrated successful conjugation of the DMPA ligand to the protein using EDC coupling without inducing cross-linking (Table 1, entry 2). On each OVA protein, there are in total 35 basic groups and 47 acidic groups, and the modified OVA showed an average molecular weight (MW) increase of 920 Da, which indicates 9 DMPA ligands conjugated per OVA protein (Figure 1B) based on surface accessibility. After modification, OVA-DMPA exhibited an average hydrodynamic diameter of 4.6 ± 0.8 nm, which is slightly increased compared to native OVA’s 4.2 ± 0.8 nm (Figure 1C). The surface zeta potential of OVA-DMPA shifted from the native acidic protein −8.3 ± 0.8 mV to the basic protein 21.1 ± 4.7 mV (Figure 1D), mainly due to the consumption of negatively charged carboxylic groups on the protein and the addition of positively charged amine groups from the ligand DMPA. Characterization by analytical ultracentrifugation (AUC) of both native and modified OVA (Figure 1E) provided us with additional protein polydispersity and aggregation state. As shown in the result curves, both native and modified OVA-DMPA displayed only one peak, indicating only monomers are observed (as only one peak is shifted from the original sedimentation coefficient), which indicates ligands were covalently bound to the protein but did not cross-link two proteins together or cause any unwanted aggregation as these events would have resulted in additional peaks. Furthermore, the width of the peak for modified OVA did not change much compared to native OVA protein, revealing that ligand DMPA was conjugated to the surface of the protein quite uniformly. Modified OVA-DMPA displayed a peak slightly left-shifted compared to the native protein, mainly due to the whole protein particle density decreasing after modification, resulting in slower sedimentation. Investigation into whether this type of protein modification will cause protein structure change by circular dichroism (CD) spectroscopy analysis of both native and modified OVA (Figure 1F) indicates that modification with ligand DMPA does not significantly change the protein’s α-helix and β-sheet structure.

### Enhanced Antigen Presentation by Direct Cytosolic Delivery of OVA-DMPA

**Figure 2:**
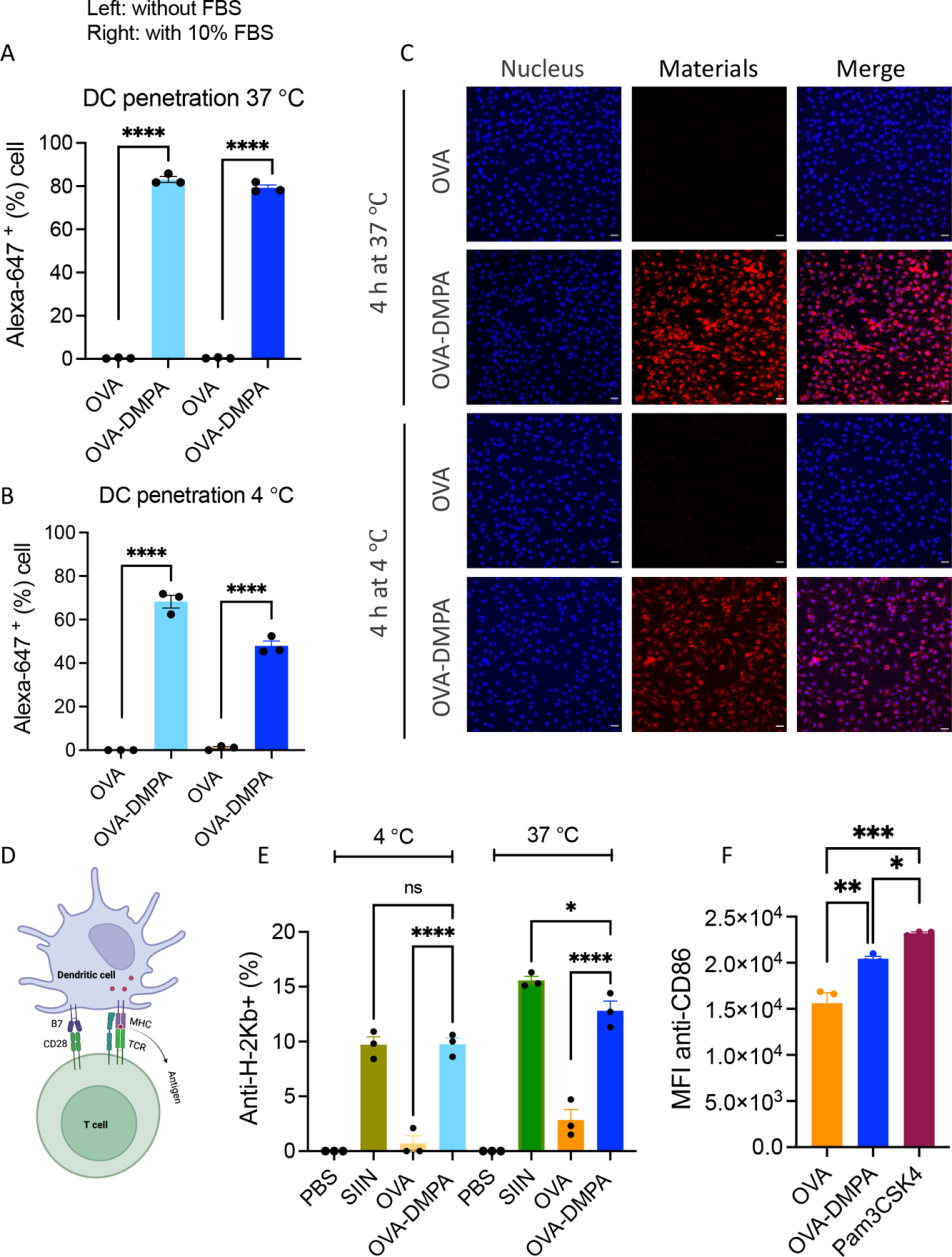
Enhanced cytosolic delivery of antigen protein OVA after surface modification. (A) Antigen presenting cells DCs cell penetration of native OVA and modified OVA-DMPA at 37 °C for 4 h with and without 10% FBS influence. (B) DCs cell penetration of native OVA and modified OVA-DMPA at 4 °C for 4 h with and without 10% FBS influence while endocytosis is inhibited at this low temperature. (C) Confocal imaging visualization of antigen protein uptake by DCs both at 37 and 4 °C for 4 h. Scale bar: 30 μm. (D) Schematic illustration of how antigens in DCs are presented to T cells for further adaptive immune response. (E) Antigen presentation efficiency of native and modified antigen protein both at 37 and 4 °C. (F) DCs maturation stimulated by modified OVA-DMPA, mean fluorescence intensity (MFI) of DC expressing maturation maker CD86. Statistics: *P < 0.05; ** *P* < 0.01; *** *P* < 0.001; **** *P* < 0.0001.

OVA, as a model antigen for immunization studies, has been widely used. However, protein antigens alone could not induce robust immune responses due to their poor permeabilization into cell membranes and are typically unable to escape from endosomes into the cytoplasm.^40^ By modifying the OVA protein by chemically conjugating an arginine mimicking ligand DMPA on the protein surface, we aim to enhance the protein cytosolic delivery efficiency. First, we evaluated the modified OVA-DMPA’s cell penetration capability. 10 μg of native OVA protein and modified OVA-DMPA labeled with an equivalent amount of fluorescent dye Alexa Fluor 647 were added to dendritic cells (DCs) with and without 10% FBS, both at 37 and 4 °C. Four hours later, cells were harvested and quantitatively analyzed by flow cytometry of their penetration efficiency. From Figure 2A,B, we can see that at both temperatures, 37 and 4 °C, modified OVA-DMPA exhibited superior cell penetration ability compared to native OVA. At 4 °C, energy-dependent endocytosis is inhibited, yet modified OVA-DMPA displays cell uptake comparable to the one observed at 37 °C. The cell penetration was further examined by confocal imaging visualization. In line with the flow cytometry results, the native protein OVA-DMPA exhibited a substantially higher level of antigen internalization compared to the native protein OVA at both 37 and 4 °C (Figure 2C). Furthermore, OVA-DMPA showed comparable uptake in 10% FBS to a standard FBS-free incubation, indicating little influence of serum proteins.

To successfully trigger the adaptive immune response, antigens need to be further presented to the downstream T cells. DCs first internalize the antigen protein into the cytoplasm; then, the protease will digest it into peptides of 8–12 amino acids in length. The antigen peptide will be presented to the TCR of T cells through the MHC-antigen complex to TCR and B7 to CD28 costimulation (Figure 2D); thus, evaluation of antigen-presentation efficiency could be a good indicator for cell cytosolic delivery of antigen proteins. To examine the antigen presentation efficiency of native antigen OVA and modified OVA-DMPA, 100 μg of each native and modified antigen protein was added to DCs. The K^b^-restricted OVA-derived epitope, SIINFEKL (SIIN), has been reported to facilitate efficient MHC-I presentation through cross-presentation. Here, PBS was added as a negative control group, and antigen peptide SIIN 2.2 nmol (corresponding to 100 μg of OVA) was added to DCs as a positive control. Figure 2E showed that modified antigen OVA-DMPA exhibited significantly higher antigen presentation efficiency compared to native antigen OVA, both at 37 and 4 °C, and compared to the positive control epitope peptide SIIN, OVA-DMPA showed a comparable level as SIIN at 37 °C and no significant difference at 4 °C. To visualize the antigen presentation, we also performed confocal imaging of native OVA and modified OVA-DMPA at different time points, 6, 24, and 48 h, both at 37 and 4 °C (Figure S1). At 6h, modified OVA-DMPA was already rapidly internalized into DCs and reached peak accumulation around 24h for antigen presentation, and at 48h, antigen fluorescence intensity was slightly decreased compared to 24h. Besides, the internalization displayed a diffusion pattern in DCs, indicating direct cytosolic delivery. At 4 °C, OVA-DMPA displayed a slightly lower but still comparable level of cell cytosolic internalization compared to that at 37 °C.

To investigate whether OVA-DMPA can stimulate immature DCs into mature DCs for enhancing antigen presentation, we incubated 100 μg each of native OVA and modified OVA-DMPA with immature DCs for 48 h, and 0.56 nmol of TLR1 and TLR2 agonist Pam_3_CSK_4_ was used as a positive control. Figure 2F shows that modified OVA-DMPA displayed higher immunostimulation compared to native OVA, which potentially can enhance the antigen presentation efficiency, but was not as strong as the professional immuno-agonist Pam_3_CSK_4_. Lastly, to investigate whether this ligand modification on proteins causes some cytotoxicity, we performed an MTS cytotoxicity assay. Various concentrations of native OVA and modified OVA-DMPA were tested on DCs, and from Figure S2, we can see over 90% of cells are viable in the relevant concentration range (100 μg/mL), and OVA-DMPA and native OVA did not show a significant difference, which indicates the biocompatibility of this ligand modification strategy on proteins for intracellular delivery.

### Enhanced Cytosolic Delivery of Various Proteins from Acidic to Basic

**Figure 3:**
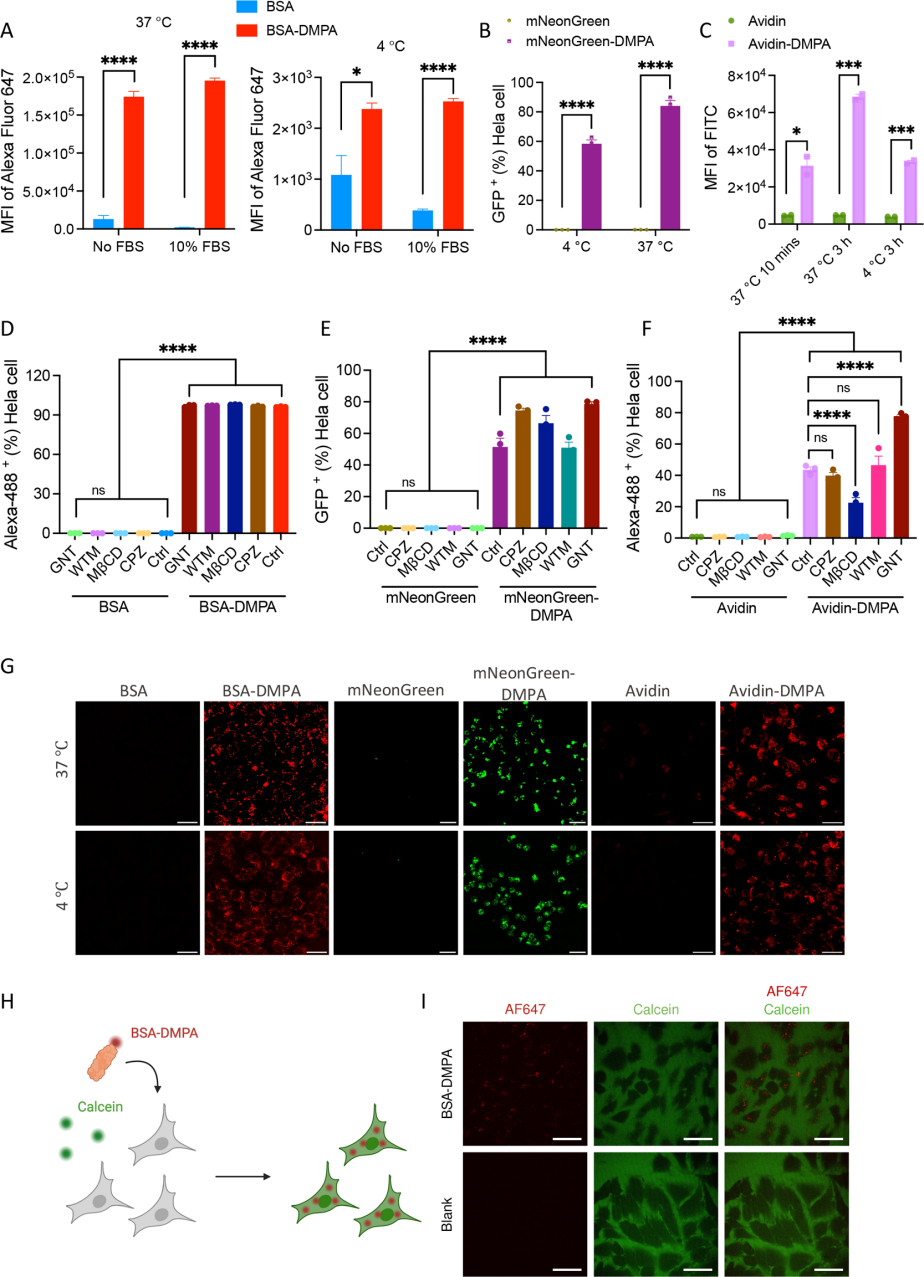
Enhanced cytosolic delivery of various proteins, BSA, mNeonGreen, and Avidin after surface modification by ligand DMPA. (A) HeLa cell penetration of dye labeled native BSA and modified BSA-DMPA at 37 and 4 °C for 4 h with and without 10% FBS influence. (B) HeLa cell penetration of autofluorescence protein mNeonGreen and modified mNeonGreen-DMPA at 37 and 4 °C for 4 h without FBS. (C) Vero cell penetration of dye labeled Avidin and modified Avidin-DMPA at 37 °C for 10 min and 3 h, and at 4 °C for 3 h all without FBS. (D–F) Inhibition of endocytic transport by chlorpromazine hydrochloride (CPZ), methyl-β-cyclodextrin (MβCD), wortmannin (WTM), genistein (GNT) for native BSA and modified BSA-DMPA, native mNeonGreen and modified mNeonGreen-DMPA, native Avidin and modified Avidin-DMPA on HeLa cell cytosol internalization. (G) Confocal imaging visualization of three different proteins BSA, mNeonGreen, and Avidin with and without surface modification cell uptake both at 37 and 4 °C for 4 h. Scale bar: 30 μm. (H) Schematic illustration of the calcein permeabilization experiment: HeLa cells are incubated with calcein in the presence of BSA-DMPA at 37 °C for 3 h, and imaged for calcein cytosol leak as an indication of membrane damage. (I) Representative confocal microscopy images of the calcein permeabilization experiment performed on HeLa cells incubated with BSA-DMPA. Scale bar: 50 μm. Statistics: *P < 0.05; ** *P* < 0.01; *** *P* < 0.001; **** *P* < 0.0001.

To check the ligand DMPA conjugating strategy’s versatility in enhancing protein direct cytosolic delivery efficiency, we selected five different proteins that have varying pI from acidic (4.5) to close to neutral (6.3) to basic (10.5) and molecular weights from 12.2 to 67 kDa: OVA, BSA, mNeonGreen, Cyto *C*, and Avidin. We optimized the chemical modification protocol to cationize these five proteins based on the number of surface accessible acidic residues available for conjugation, ultimately producing proteins with a uniform surface charge. In this section, we mainly discuss three inert proteins (BSA, mNeoGreen, and Avidin). After careful purification and characterization of their physicochemical properties (Table 1, entries 4, 6, and 10) and their protein secondary structures (Figure S3), we first assessed their cell penetration efficiency. 10 μg of native protein and modified protein-DMPA labeled with an equivalent amount of fluorescence dye, Alexa Fluor 647 for BSA, FITC for Avidin, and mNeonGreen using its autofluorescence, were added into cells both at 37 and 4 °C. Cells were harvested and quantitatively analyzed by flow cytometry. From Figure 3A, we observed that at both 37 and 4 °C, BSA-DMPA exhibited significantly higher cell internalization in HeLa cells compared to native BSA, and 10% FBS during the incubation did not affect the cell uptake. Similar effects were also observed for the neutral protein mNeonGreen-DMPA and the basic protein Avidin-DMPA; they displayed superior cell penetration compared to native mNeonGreen and Avidin, both at 37 and 4 °C (Figure 3B, C). Especially for Avidin-DMPA, rapid cell internalization was observed after only 10 min of incubation at 37 °C, and continued to increase with time, while native Avidin maintained relatively low levels over time and temperatures (Figure 3C).

To understand how the ligand chemistry on proteins affects their cell penetration, we modified BSA with ligands featuring different hydrophobicities and terminal group charges (Scheme S1). Besides the ligand DMPA discussed above, we also investigated the ligands *N*,*N*′-dimethyl-1,6-hexanediamine (DMHA) and *N*,*N*′-dimethyl-1,12-diaminododecane (DMDA) containing 6 methylene (6-C) and 12-C long aliphatic chains to investigate the role of hydrophobicity. Ligands 5-aminopentanesulfonic acid (APS) and cadaverine (CAD) were used to investigate the role of an anionic end group and a primary amine end group, respectively. Products were synthesized with the same protocol as the ligand DMPA protein modification and were carefully characterized (Table S1). Cell penetration was measured on HeLa cells at both 37 and 4 °C. From the results, we observed that at 37 °C, increased ligand hydrophobicity did not influence the cell penetration efficiency, while at 4 °C, increased ligand hydrophobicity facilitated direct cytosolic delivery of proteins (Figure S4A,B). Besides, we also observed almost no cell penetration of BSA-APS, suggesting that cationic charge is one of the key factors for protein direct cytosolic penetration (Figure S4C,D). The reason for not using the primary amine terminal group is for cytotoxicity considerations.

To further investigate whether this exceptional penetration behavior of these three modified proteins is due to variations in endocytosis, we applied four typical endocytic transport inhibitors: chlorpromazine hydrochloride (CPZ) targeting clathrin-mediated endocytosis (CME),^41^ methyl-β-cyclodextrin (MβCD) targeting lipid rafts/cholesterol-enriched microdomains/caveolae,^42^ wortmannin (WTM) targeting phosphoinositide 3-kinases (PI3Ks),^43^ and genistein (GNT) targeting multiple tyrosine kinases.^44^ After treatment with the endocytic transport inhibitors, dye-labeled native and modified proteins were added to cells for incubation, followed by quantitative analysis of protein cell penetration by flow cytometry. Figure 3D–F illustrates that all modified proteins penetrate cells despite the use of inhibitors, while all unmodified proteins do not. This confirms the finding of the 4 °C experiments that modified proteins can directly penetrate the plasma membrane. However, in the cases of mNeonGreen-DMPA and Avidin-DMPA, the number of proteins found in the cytosol varies depending on the inhibitor, indicating that the protein could take alternative cellular endocytic uptake pathways besides the direct penetration.

The cell penetration was further examined by confocal imaging. Consistent with the flow cytometry results, BSA-DMPA, mNeonGreen-DMPA, and Avidin-DMPA exhibited a substantially higher level of cell internalization compared to the native proteins BSA, mNeonGreen, and Avidin, both at 37 and 4 °C (Figure 3G). Detailed images are shown in Figures S5–S7. We also imaged calcein permeabilization in the presence of BSA-DMPA with the confocal microscope, a method reported previously to visualize cell membrane damage caused by cationic molecules^20,45^ (Figure 3H). Many cationic drug delivery carriers have been shown to cause the cell-impermeable dye calcein to leak into the cytosol due to cellular membrane damage^46^; we did not observe the calcein leak induced by BSA-DMPA (Figure 3I). This indicates that the DMPA modification leads to membrane penetration without overt poration.

To further confirm that Avidin after surface modification still retains biotin binding ability, we performed the Avidin–Biotin Complex (ABC) method to native Avidin and modified Avidin-DMPA. Absorbance at 490 nm was measured, and the result showed us that both native and modified Avidin still retained strong binding ability to biotin (Figure S8), further proving that the secondary protein structure and its function are not significantly changed after modification.

We also performed an MTS cytotoxicity assay on modified proteins BSA-DMPA and Avidin-DMPA. Various concentrations of native BSA and modified BSA-DMPA, native Avidin, and modified Avidin-DMPA were tested; from Figure S9, we can see over 95% of cells are viable while the concentration is less than or equal to the highest concentration tested. Native protein and protein-DMPA did not show significant differences in their toxicity profile, which indicates the cytocompatibility of this ligand modification strategy on these proteins. Based on the above observations, this simple ligand DMPA tagging on the surface of proteins strategy showed its versatility in enhancing direct cell cytosol delivery.

### Enhanced Cell Apoptosis by Direct Cytosolic Delivery of Cyto *C*-DMPA

**Figure 4:**
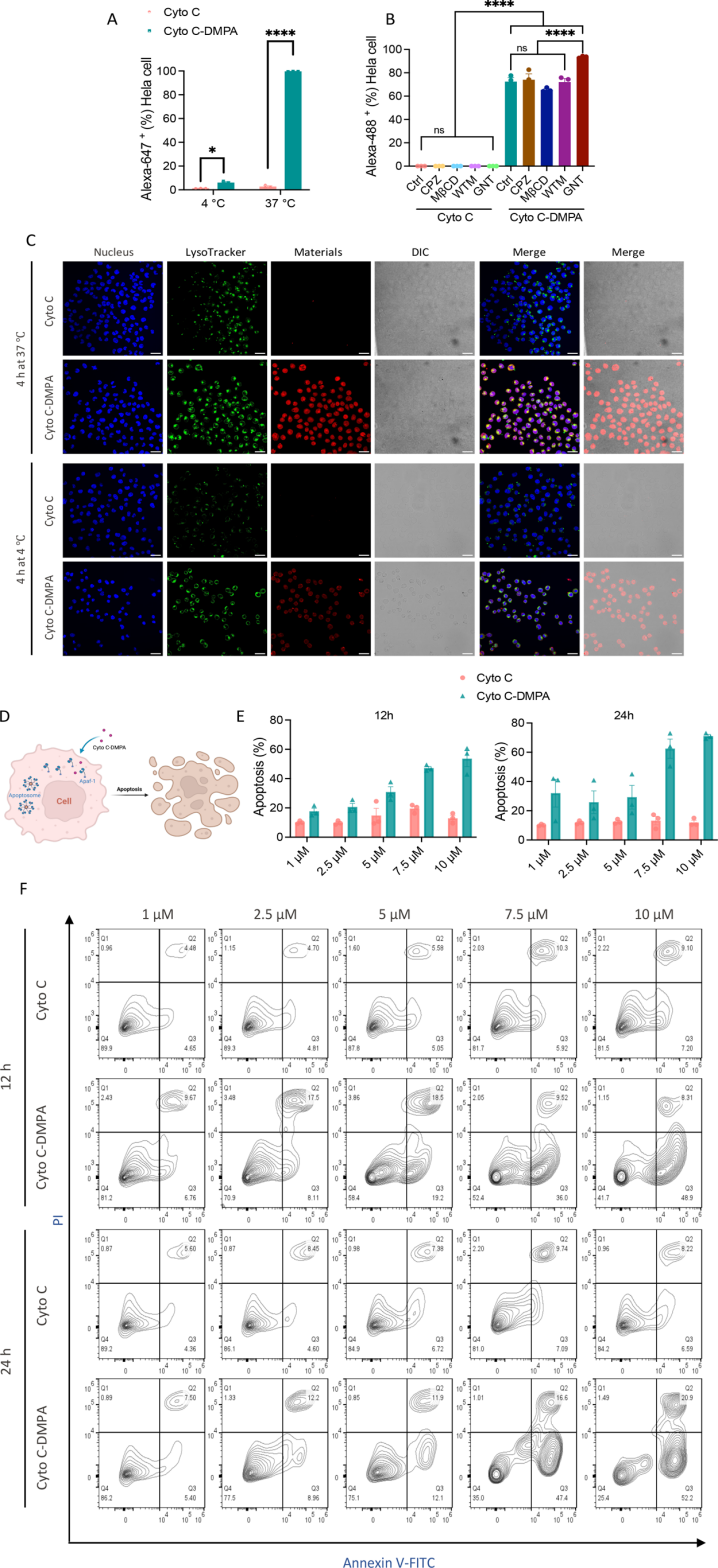
Enhanced cell apoptosis by direct cytosol delivery of Cyto *C*-DMPA. (A) HeLa cell penetration of dye labeled native Cyto *C* and modified Cyto *C*-DMPA at 37 and 4 °C for 4 h. (B) Inhibition of endocytic transport by CPZ, MβCD, WTM, GNT for native Cyto *C* and modified Cyto *C*-DMPA on HeLa cell cytosolic internalization. (C) Confocal imaging visualization of native Cyto *C* and modified Cyto C-DMPA cell uptake both at 37 and 4 °C for 4 h. Scale bar: 30 μm. (D) Schematic illustration of how protein Cyto *C*-DMPA cause cell apoptosis. (E) Quantitive percentage of HeLa cell apoptosis of native Cyto *C* and modified Cyto *C*-DMPA at various concentrations for 12 and 24 h. (F) Representative flow cytometry plots of frequencies of HeLa cell apoptosis of native Cyto *C* and modified Cyto *C*-DMPA at various concentrations for 12 and 24 h. Statistics: *P < 0.05; ** *P* < 0.01; *** *P* < 0.001; **** *P* < 0.0001.

Cytochrome *C* (Cyto *C*) plays a key role in cell apoptosis, in fact, during cell apoptosis, Cyto *C* is released into the cytoplasm, where it binds and activates the apoptotic protease activating factor-1 (Apaf-1), allowing its binding to ATP and the formation of the ring-like apoptosome.^47,48^ (Figure 4D) Fluorescent conjugates of annexin V are commonly used to identify apoptotic cells, especially as indicators of intermediate stages of apoptosis. In this study, to evaluate the cytosolic delivery efficiency of modified Cyto *C*-DMPA, we first examined its cell penetration capability. By applying the same chemical modification reaction to the protein Cyto *C*, we can successfully obtain Cyto *C*-DMPA. Characterizations of the modified proteins are presented in Table 1. Protein secondary structures were also analyzed compared to native Cyto *C* (Figure S10). After confirming their physicochemical properties, 10 μg of native protein and modified Cyto *C*-DMPA labeled with an equivalent amount of fluorescence dye Alexa Fluor 647, were added into cells both at 37 and 4 °C. Cells were harvested and quantitatively analyzed by flow cytometry. From the results, we can see that at 37 °C, modified Cyto *C*-DMPA showed significantly higher penetration in HeLa cells compared to native Cyto *C*, while at 4 °C, Cyto *C*-DMPA showed only a slight increase in penetration compared to Cyto *C* (Figure 4A). One possible reason is that this protein, modified or not, mainly went through endocytosis, but another possibility is that most of Cyto *C*-DMPA entered the cytosol of HeLa very quickly at 4 °C, which caused the majority of cells’ apoptotic death. A complementary study with endocytic transport inhibitors CPZ, MβCD, WTM, and GNT was further performed to investigate the cell penetration mechanism. Dye-labeled native and modified Cyto *C* were added into cells for incubation after treatment with these four endocytic transport inhibitors, and quantitative analysis of protein cell penetration by flow cytometry was performed. From Figure 4B, we can see that all inhibitors cannot stop modified Cyto *C*-DMPA’s cell penetration, which indicates that, after ligand modification, proteins might directly pass through the plasma membrane into the cytosol. Thus, we conclude that Cyto *C*-DMPA entered HeLa cells through direct cytosolic fusion efficiently both at 37 and 4 °C; the low frequency of cell penetration at 4 °C observed should be mainly due to the combined effect of cell apoptosis plus cell stress.

Cell penetration was further examined by confocal imaging. Consistent with the flow cytometry results, Cyto *C*-DMPA exhibited a substantially higher level of cell internalization compared to the native proteins Cyto *C*, both at 37 and 4 °C (Figure 4C). Cyto *C*-DMPA exhibited a nice diffusion pattern inside the whole cell, even colocalized with the nucleus, both at 37 and 4 °C. This phenomenon further demonstrated that Cyto *C*-DMPA penetrated HeLa cells through direct cytosolic delivery. Co-localization of penetrated proteins with the nucleus is probably due to their small molecular weight.^49^ Besides, at 4 °C, Cyto *C*-DMPA also showed less density of cells under confocal imaging compared to that at 37 °C, which is consistent with the observations in flow cytometry. Cell morphologies under the bright field of Cyto *C*-DMPA at 4 °C clearly displayed cell apoptosis due to cytosolic delivery of Cyto *C* and cell stress due to the low temperature.

To quantitatively evaluate cell apoptosis caused by cytosolic delivery of Cyto *C*-DMPA, we performed an “Annexin V Apoptosis Assay”. Both native Cyto *C* and modified Cyto *C*-DMPA at various concentrations were incubated with HeLa cells for 4 h at 37 °C. Cells were then washed and cultured with normal cell medium with 10% FBS for 12 and 24 h to observe apoptosis at different time points. Cells were then harvested and stained with Annexin V and propidium iodide (PI) for flow cytometry analysis. Native Cyto C showed an almost constant fraction of apoptotic cells independent of incubation time (12 and 24 h) and protein concentration. Compared with the cells treated with native Cyto *C*, the cells treated with Cyto *C*-DMPA showed a significantly higher fraction of apoptotic cells (Figure 4E). At 12 h incubation, a clear dependence on protein concentration was observed; it was also observed at 24 h, but in a less clear way. Overall, the effect became larger after longer incubation. From representative flow cytometry plots of frequencies of HeLa cell apoptosis of Cyto *C* and Cyto *C*-DMPA (Figure 4F), we can clearly observe the cell density migration from Annexin V^–^PI^–^ (healthy cells) to Annexin V^+^ or Annexin V^+^PI^+^ (apoptotic cells) of Cyto *C*-DMPA according to increasing concentrations, both incubation for 12 and 24 h, suggesting the increasing cell apoptotic level. All of these results suggest that Cyto *C*-DMPA was efficiently delivered into the cell cytoplasm and induced cellular apoptosis.

## Conclusions

Here we report a simple, versatile, and highly efficient protein modification strategy for intracellular delivery via conjugation of the cationic and hydrophobic ligand DMPA. This strategy was applied to successfully deliver all negatively, neutral, and positively charged proteins listed in this work into different cell lines. The bioactivities of delivered proteins were well maintained after this surface modification. We also tested larger proteins such as Cas13a nuclease (143.7 kDa) (Figure S11), which showed successful cell cytosolic penetration, but the RNase activity was hampered (Figure S12). In the future, a preprotection of functional sites could be envisioned before performing the ligand modification. In this work, the delivery potency of chemically tagging DMPA on the surface is mainly attributed to the conjugation of an arginine-mimicking cationic ligand with a small hydrophobic domain, which can help the protein enter cells in an energy-independent way known as direct translocation. This study provides a new, yet simple strategy for efficiently delivering proteins directly to the cell cytosol and offers an important new insight into the design and development of novel arginine-rich cell-penetrating peptide mimetic molecules to enable protein transduction.

## Experimental (Methods and Materials)

### Materials

Protein ovabumin (OVA), bovine serum albumin (BSA), cytochrome *C* (Cyto *C*), and peptide SIINFEKL were purchased from Sigma-Aldrich (Missouri, United States). Protein Avidin is from Santa Cruz Biotechnology (Texas, United States). Protein mNeonGreen was home-synthesized in the EPFL protein expression facility. Protein Cas13a was purchased from Genscript Biotech (New Jersey, United States). Chemicals 1-Ethyl-3-(3-(dimethylamino)propyl) carbodiimide hydrochloride (EDC), *N*,*N*′-dimethyl-1,3- propanediamine (DMPA), *N*,*N*′-dimethyl-1,6-hexanediamine (DMHA), *N*,*N*′-dimethyl-1,12-diaminododecane (DMDA), 5-aminopentanesulfonic acid (APS), cadaverine (CAD), calcein, and 4% PFA solution were purchased from Sigma-Aldrich (Missouri, United States). MES buffer 4.7, Alexa FluorTM 647 *N*-hydroxysuccinimide (NHS) ester, NHS-fluorescein (5/6-carboxyfluorescein succinimidyl ester), and Alexa Fluor 488 NHS Ester (Succinimidyl Ester). Dead cell apoptosis kits with annexin V for flow cytometry were purchased from Thermo Fisher Scientific (Ecublens, Switzerland). DAPI solution, ProLongTM diamond antifade mountant, and Hoechst 33342 were purchased from BioLegend (San Diego, California, United States). Endocytosis inhibitors chlorpromazine (CPZ), methyl-β-cyclodextrin (MβCD), Wortmannin (WTM), and Genistein (GNT) were purchased from Sigma-Aldrich (Missouri, United States). Antibodies for fluorescence-activated cell sorting (FACS), including anti-CD16/32 (Clone: 93) and anti-CD86 (Clone: GL-1), antimouse H-2K^b^ (AF6-88.5), were purchased from BioLegend (San Diego, California, United States). CellTiter 96 AQueous One Solution Cell Proliferation Assay (MTS) was purchased from Promega (Wisconsin, U.S.). LysoTracker Red DND-99, Hoechst 33342, and ProLong diamond antifade mountant were purchased from Life Technology (California, U.S.). Cell culture-related materials such as medium DMEM, RPMI 1640, fetal bovine serum (FBS), penicillin-streptomycin (P/S) (10,000 U/mL), Trypsin-EDTA (0.25%), and PBS pH 7.4 (1×) were purchased from Life Technology (California, United States). Unless otherwise noted, all chemical and biological reagents were used as received. All solvents purchased were reagent grade.

### Cell Line

Vero cells (African green monkey fibroblastoid kidney cells) were purchased from ATCC (CCL-81) and cultured in DMEM, high glucose, GlutaMAX Supplement, pyruvate supplemented by fetal bovine serum (FBS, 10%), Penicillin (100 U mL^–1^), and Streptomycin (100 μg mL^–1^). Cells were cultured in a humidified atmosphere with 5% CO_2_ at 37 °C.

DC2.4 cells were a kind gift from Prof. Kenneth L. Rock (Dana-Farber Cancer Institute, Inc., DFCI). It was cultured in RPMI 1640, GlutaMAX Supplement medium with fetal bovine serum (FBS, 10%), penicillin (100 UmL^–1^), and streptomycin (100 μgmL^–1^). Cells were cultured in a humidified atmosphere with 5% CO_2_ at 37 °C.

HeLa cells were a kind gift from Maartje Basting (École Polytechnique Fédérale de Lausanne, EPFL). It was cultured in DMEM, GlutaMAX Supplement, with fetal bovine serum (FBS, 10%), penicillin (100 U mL^–1^), and streptomycin (100 μg mL^–1^). Cells were cultured in a humidified atmosphere with 5% of CO_2_ at 37 °C.

### Instruments

Protein–ligand products’ size and surface zeta potential were characterized by dynamic light scattering (DLS) and zeta potential on Malvern NanoZS (Worcester, UK). Matrix-assisted laser desorption/ionization time-of-flight mass spectra (MALDI-TOF-MS) were acquired on an Autoflex Speed instrument (Bruker, Billerica, Massachusetts, USA). Protein structure integrity was measured with a circular dichroism spectrometer (CD), Chirascan V100 from Applied Photophysics (Leatherhead, UK). Analytical ultracentrifugation (AUC) was performed by using Beckman Optima XL-A, An-60 Ti rotor (California, United States). All of the flow cytometry data were acquired using an Attune NxT flow cytometer (Thermo Fisher Scientific). Confocal fluorescent microscopy images were acquired with the Inverted Leica DMi8 with 40× and 63× oil objectives (Leica, Wetzlar, Germany) and the Nikon Spinning Disk CSU W1 with a 60× oil objective (Nikon, Japan). The fluorescence intensity of samples was measured with a Varioskan Lux microplate reader (Thermo Fisher Scientific) and a Biotek Synergy H1 microplate reader (Biotek, USA).

### Methods

#### Engineering of Proteins via Conjugation with Ligands

To prepare the modified proteins conjugated with ligand DMPA, three solutions were prepared separately first. Solution 1: proteins BSA or Avidin or Cytochrome C or OVA or mNeonGreen was dissolved in MES pH = 4.7 buffer (30 mg into 7.5 mL buffer with concentration 4 mg/mL); Solution 2: ligand DMPA was weighted 600 mg, and dissolved in 1–2 mL miliq water, adjust pH to ∼7.0 by adding 1 M HCl, top-up the final volume into 15 mL with miliq water, final ligand concentration is 40 mg/mL. Solution 3: EDC 300 mg was dissolved in 7.5 mL of MES pH = 4.7 buffer, and the final concentration of EDC solution is 40 mg/mL. In this reaction, the mass ratio of protein, ligand, and EDC is fixed into protein:ligand:EDC = 1:20:10. Mix solutions 1, 2, and 3 together with magnetic stirring at around 600 rpm overnight at room temperature. Purification of the final products was carried out by using an Amicon filter tube with a molecular cutoff of 30 kDa for BSA, OVA, and Avidin protein and 10 kDa for Cytochrome *C* and mNeonGreen protein; miliq water was top-up to around 10 mL for washing at least 5 times with centrifugation under 5000 rpm speed for 5 min.

Other ligands DMHA, DMDA, CAD, and APS conjugation were applied in the same protocol as that above.

#### Characterization of Proteins Conjugated with Ligands

##### DLS

All purified protein–ligand product solutions were in miliq water with a concentration of 1 mg/mL. An Eppendorf disposable cuvette with an absorbance range of 220–1600 nm was used. A 100 μL volume was put in the cuvette and measured by the instrument Malvern NanoZS with condition manual scan for 10 runs under room temperature.

##### Zeta Potential

All purified protein–ligand product solutions were in milliliter water with 1 mM KCl in concentration 1 mg/mL. Malvern disposable folded capillary cells DTS1070 was used for the measurement in the instrument Malvern NanoZS.

##### Mass Spectrum

MALDI-TOF analyses were performed on an Autoflex Speed time-of-flight mass spectrometer (Bruker Daltonics, Bremen, Germany) equipped with a Bruker smartbeamTM-II laser (355 nm wavelength) and operated in the linear positive mode. Ion source 1 was set to 19.6 kV, ion source 2 was set to 17.5 kV, Pulsed Ion Extraction was set to 28 kDa and the mass range for detection was setup based on the specific proteins. Spectra were acquired using flexControl version 3.4. The three-layer method was used to spot the samples. Briefly, 1 μL of sinapinic acid (SA, Merck) matrix solution at 20 mg/mL in acetone was deposited on each spot of an MTP 384 ground steel BC target plate (Bruker, DE) and allowed to dry again at room temperature, forming a very thin first layer of matrix. The sample was centrifuged at 10,000 × *g* for 2 min, and 1 μL of the supernatant was spotted on the target and allowed to dry at room temperature. After drying, 1 μL of SA matrix solution at 10 mg/mL in 50% acetonitrile, 47.5% water, and 0.1% trifluoracetic acid was applied to each spot and allowed to dry again at room temperature. Each spectrum was collected as a minimum of 2000 shots. For each measurement, the spectra were manually processed using flexAnalysis 3.4 Compass 1.4 (Bruker Daltonics, DE). For calibration, 0.5 L of Bruker Protein Standard II was deposited by using the same method as the supernatant.

##### Circular Dichroism (CD)

CD spectroscopy was used to analyze the effect of the cationization process on the protein secondary structure. CD experiments were performed on an Applied Photophysics Chirascan V100 Spectropolarimeter with quartz cells (*l* = 0.1 cm). Spectra were obtained from aqueous solutions (0.1–0.2 mg mL 1 in 50 mM phosphate buffer), and data was collected with 1 nm steps between 260–180 nm and 2 s integration time per step. A minimum of three spectra was recorded for static scans at 25 °C.

##### Analytical Ultracentrifugation (AUC)

AUC was performed by using a Beckman Optima XL-A, An-60 Ti rotor. All sample solutions were prepared freshly in PBS buffer to obtain final solutions that had 0.5–1.0 OD (optical density) absorbance at 280 nm in AUC cells (double sector titanium centerpieces with quartz windows; the optical path length is 1.2 cm). All measurements were made at 20 °C, 50,000 rpm. (with a radial step size of 0.003 cm) with sufficient duration to ensure complete sedimentation. Data ranges from 50 to 100 scans were chosen to represent the whole transporting process.

#### Fluorescent Labeling of Native Proteins and Modified Protein–Ligand Products

To evaluate modified protein–ligand materials’ cell penetration ability, we prepared native proteins and their corresponding modified protein (-DMPA, -DMHA, DMDA, -APS, and -CAD) samples with a concentration of 5 mg/mL. Fluorescent dye Alexa Fluor 647 NHS ester (10 mg/mL in anhydrous DMSO) is prepared for labeling OVA, BSA, Cyto *C*, and their corresponding modified proteins; for mNeonGreen native and its modified one, we utilize their auto fluorescence (GFP channel), and NHS-Fluorescein (5/6-carboxyfluorescein succinimidyl ester) (10 mg/mL in anhydrous DMSO) was used to label protein Avidin and Avidin-DMPA. Native protein or modified protein solutions were added with fluorescent dye with equal stoichiometry and then shaken with an Eppendorf ThermoMixer at 25 °C (600 rpm, 30 min). The “Labeled” mixture was used for the next step without purification. For in vitro studies, 10% of NH_2_ groups on the proteins were fluorescently labeled for flow cytometry assays, and 20% of NH_2_ groups on the proteins were fluorescently labeled for confocal fluorescent microscope imaging. The dye-labeled mixture was further diluted 5 times into 1 mg/mL with PBS 1×.

For the endocytosis inhibition cell penetration assay, all the native protein and modified protein–ligand (except mNeonGreen and mNeonGreen-DMPA) were labeled with Alexa Fluor 488 NHS Ester (Succinimidyl Ester) (10 mg/mL in anhydrous DMSO). Native protein or modified protein solutions were added with fluorescent dye with equal stoichiometry and then shaken with an Eppendorf ThermoMixer at 25 °C (600 rpm, 30 min). The “Labeled” mixture was used for the next step without purification. In this study, 10% of the NH_2_ groups on the proteins were fluorescently labeled for flow cytometry assays.

#### In Vitro Cell Penetration Assay

For protein BSA, mNeonGreen, Cyto C, and their ligand-modified proteins, HeLa cells were seeded at a density of 0.2 × 10^6^/well into a 12-well plate 1 day in advance. For Avidin and Avidin-DMPA, Vero cells were seeded at a density of 0.2 × 10^6^/well into a 12-well plate 1 day in advance. For the OVA and the OVA-DMPA samples, DC2.4 cells were seeded at a density of 0.15 × 10^6^/well into a 12-well plate 1 day in advance. Fluorescence dye-labeled protein and protein–ligand with final concentration 1 mg/mL were added 10 μg each well into the cells, then incubated with cells at 37 and 4 °C (BSA, mNeonGreen, Cyto C and their modified proteins with HeLa cells for 4 h, OVA and OVA-DMPA samples with DC2.4 cells for 4 h, and Avidin and Avidin-DMPA samples with Vero cells for 10 min and 3 h) with 1 mL of DMEM medium (HeLa and Vero cells) or RPMI 1640 medium (DC2.4 cells) without FBS (among them, OVA, OVA-DMPA and BSA, BSA-DMPA were also tested cell penetration with 10% FBS). Afterward, cells were washed with PBS 1× twice, detached, harvested, and washed with FACS buffer (PBS 1× containing 0.2% BSA) 200 μL × 2. The cells were stained with and resuspended in a DAPI solution (0.1 μg/mL, 200 μL) for flow cytometry analysis.

#### Inhibition of Endocytic Transport for the Evaluation of Internalization Mechanism

HeLa cells were seeded at the density of 0.15 × 10^6^ cells/well in 12 well-plates 12 h before the experiment, and the culture was maintained in Dulbecco’s modified Eagle medium (DMEM) supplemented with 10% fetal bovine serum, 1% penicillin-streptomycin at 37 °C in a humidified atmosphere of 5% CO_2_. After 12 h, the medium was aspirated. Cells were washed with PBS once, followed by the treatment of four endocytosis inhibitors dissolved in serum-free medium at the following concentrations: chlorpromazine hydrochloride (CPZ, 10 μg/mL), methyl-β-cyclodextrin (MβCD, 6.7 mg/mL), wortmannin (WTM, 10 μg/mL), and genistein (GNT, 100 μg/mL). The control group was treated with the same volume of the serum-free medium without any inhibitor. Treated cells were preincubated at 37 °C for 30 min. The experiment was performed in triplicate. After incubation, the medium was aspirated, and the cells were washed with PBS twice. Then, they were treated with the proper amount (20 μg protein/well) of native proteins and modified proteins (protein–ligand) labeled with Alexa-Fluor-488 in serum-free DMEM for another 4h. Cells were washed thrice with PBS and incubated at 37 °C for 24h in DMEM containing 10% FBS. The next day, cellular nuclei were stained with DAPI, and a fluorescence-activated cell sorting (FACS) flow cytometer was used to determine the uptake level.

#### OVA Modification Influence on Antigen Presentation

In this assay, DC2.4 cells were seeded at a density of 0.1 × 10^6^ cells/well in 12-well plates around 12 h in advance. Protein OVA and modified OVA-DMPA 100 μg each were added into cells with 1 mL of RPMI 1640 medium without FBS for 3 h. PBS 1× was added as a negative control group, and antigen peptide SIINFEKL 2.2 nmol (corresponding to 100 μg OVA) was added into cells as a positive control. All samples are triplicated and incubated with cells both at 37 and 4 °C for 3 h. After incubation, we removed the material-containing medium, washed the cells with PBS 1× twice, added 1 mL of RPMI 1640 complete medium (with 10% FBS), and let it incubate at 37 °C for 48 h. Afterward, cells were washed with PBS 1× twice, detached, harvested, and washed with FACS buffer (PBS 1× containing 0.2% BSA) 200 μL × 2. The cells were first stained with anti-CD16/32 at 4 °C for 15 min and then stained with PE-anti-H-2Kb at 4 °C for 20 min, washed with FACS buffer (200 μL × 2), and resuspended in a DAPI solution (0.1 μg mL^–1^, 200 μL) for flow cytometry analysis.

#### OVA Modification Influence on DC Activation

Immature DCs were plated in 12-well plates with a seeding density of 0.1 × 10^6^ cells/well in RPMI 1640 complete medium (1 mL), 1 day in advance. Sample OVA and modified OVA-DMPA 100 μg each) were added to the cells the next day. The TLR1 and TLR2 agonist Pam_3_CSK_4_ (0.56 nmol) was added as a positive control. All samples are triplicated in this assay. After 48 h incubation, DCs were first washed by PBS 1× once, detached, and harvested; they were further washed with FACS buffer (200 μL × 2), incubated with anti-CD16/32 at 4 °C for 15 min, then stained with BV510-anti-CD86 at 4 °C for 20 min, followed by washing with FACS buffer (200 μL × 2), and resuspended in a DAPI solution (0.1 μg mL^–1^, 200 μL) for flow cytometry analysis.

#### Cytochrome *C* (Cyto *C*) Modification Influence on Cell Apoptosis

HeLa cells were seeded at a density of 7.5 × 10^4^ cells/well in 24 well-plates 12 h before the experiment, and the culture was maintained in Dulbecco’s modified Eagle medium (DMEM) supplemented with 10% fetal bovine serum, 1% penicillin-streptomycin at 37 °C in a humidified atmosphere of 5% CO_2_. After 12 h, the medium was aspirated. Cells were washed with PBS once, followed by the treatment of bare Cyto-*C* and modified Cyto-*C*-DMPA at the concentrations of 1, 2.5, 5 μM, 7.5 μM, and 10 μM for 4h at 37 °C in serum-free culture medium. The experiment was performed in triplicate. After incubation, cells were washed with PBS 1× twice, and the culture medium was replaced with DMEM containing 10% FBS and incubated for 12 and 24 h to observe apoptosis at different time points. Following incubation, cells were washed with cold cell staining buffer and then Annexin V binding buffer (BioLegend, UK) and stained with FITC annexin V and propidium iodide (BioLegend, UK) for FACS analysis, detail procedure followed by “Dead cell apoptosis kits with annexin V for flow cytometry (V13242)”.

#### Cytotoxicity Assay

Modified proteins’ cell cytotoxicity was evaluated on Vero cells, HeLa cells, and DC2.4 cells with CellTiter 96 AQueous One Solution Cell Proliferation Assay (MTS). Cells were plated around 24 h in advance in a 96-well plate with a seeding density of 2 × 10^4^/well in DMEM medium containing 10% FBS. Materials were serially diluted into DMEM medium, and each diluted sample volume was kept at 200 μL. The original cell culture medium was replaced with material containing a medium of 200 μL each well and incubated at 37 °C in the cell culture incubator for 24 h. After that, the material containing medium was removed, and the cells were washed with PBS 7.4 (1×) twice and then MTS reagents 10 μL + 90 μL DMEM serum-free medium into each well and incubated at 37 °C for 4 h. After incubation, absorbance at 490 nm was measured with a microplate reader Tecan. The cell viability ratio was calculated compared to nondrug-treated cells.

#### Confocal Fluorescent Microscope Imaging for Cell Penetration

Native proteins and their modified protein–ligand products were prepared in PBS 1× solution with a concentration of 5 mg/mL. Except for mNeonGreen and mNeonGreen-DMPA, all other samples were first labeled by the fluorescent dye Alexa Fluor 647 NHS ester or Alexa Fluor 488 NHS ester (10 mg/mL in anhydrous DMSO). The dye labeling protocol was described above. Vero cells, DC2.4 cells, or HeLa cells were seeded at a density of 0.2 × 10^6^/well into a 6-well plate 1 day in advance. A glass cover slide was put inside the well during seeding of the cells in order to let the cells grow on top of it. Next day, dye-labeled native and modified proteins with a final concentration of 1 mg/mL were added 20 μg each well into cells, then incubated cells at 37 and 4 °C with 1 mL of medium without FBS for 4 h. Afterward, cells were washed by PBS 1× twice and stained by LysoTracker Red DND-99 (125 nM) for endolysosome staining and Hoechst 33342 (10 μM) for nuclei staining in 1 mL of phenol/serum-free medium at 37 °C with CO_2_ for 1.5 h followed by PBS washing (1 mL × 2). Cells were then fixed with 4% paraformaldehyde (PFA, 500 μL) for 15 min at 37 °C, followed by PBS washing (1 mL × 2). The cells grown and stained cover slide was sealed onto a polylysine-coated glass slide with 15 μL of ProLong diamond antifade mountant. The cells were imaged with a Leica DMi8 with 40× and 63× oil objectives.

For the visualization of the visualization of the antigen presentation of OVA and OVA-DMPA antigen presentation of OVA, we incubated 100 μg each of dye-labeled OVA and the visualization of OVA-DMPA with DC2.4 cells. Cell density and glass cover slides were prepared as above-described. At set time points (6, 24, and 48 h), DCs were washed by PBS 1× twice, and stained by LysoTracker Red DND-99 (125 nM) for endolysosome staining and Hoechst 33342 (10 μM) for nuclei staining in 1 mL of phenol/serum-free medium at 37 °C with CO_2_ for 1.5 h followed by PBS washing (1 mL × 2). Cells were then fixed with 4% paraformaldehyde (PFA, 500 μL) for 15 min at 37 °C, followed by PBS washing (1 mL × 2). Cell grown and stained cover slide was sealed onto a polylysine-coated glass slide with 15 μL of ProLong diamond antifade mountant. The cells were imaged with a Leica DMi8 instrument with a 40× oil objective.

For the calcein penetration experiment, HeLa cells were seeded in an ibidi 8-well μ-Slide at a seeding density of 1.5 × 10^4^ cells/mL 24 h before the experiment. BSA-DMPA was fluorescently labeled with AlexaFluor647-NHS according to the protocol above just before the calcein BSA-DMPA coincubation. Calcein was dissolved in DMSO to a 10 mg/mL concentration as a stock solution. The cell culture media were replaced with fresh full culture media, 1 μg of labeled BSA-DMPA was added, and calcein was added to a final concentration of 75 μg/mL. The cells were incubated at 37 °C with 5% CO_2_ for 3 h, and live imaging was performed at 37 °C with 5% CO_2_ utilizing the Nikon Spinning Disk CSU W1 confocal microscope (60× oil objective).

#### RNase Activity Assay

To evaluate the function of the Cas13a protein following the modification of the cationic ligand, we assayed the RNase activities of Cas13a and Cas13a-DMPA utilizing a self-quenched RNA reporter assay. Two crRNAs were designed to target the EGFP mRNA: crGFP1 (5′-GAU UUA GAC UAC CCC AAA AAC GAA GGG GAC UAA AAC AAU UUA GUA AUU GUU CGG ACA CU-3′), crGFP2 (5′-GAU UUA GAC UAC CCC AAA AAC GAA GGG GAC UAA AAC AUU CAA CAA GAA UUG GGA CAA CU-3′), and a negative control crRNA crNeg (5′-GAU UUA GAC UAC CCC AAA AAC GAA GGG GAC UAA AAC GUA GAU CAU UGU ACG AUC UAU UA-3′) was used as a nontargeting crRNA. To investigate the RNase activity, the RNaseAlert v2 system was used, with 45 nM of proteins (Cas13a, Cas13a-DMPA, and RNase A as a positive control for RNase activity), 22.5 nM of crRNAs, and 125 nM of quenched fluorescent reporter at a total volume of 50 μL. The reaction mixture was incubated at 37 °C for 20 min on a Biotek Synergy H1 plate reader, followed by the fluorescence kinetics measurement of ex/em = 490/520 nm performed every 5 min for 2 h. Nuclease-free water was added to the assay system as the blank for the fluorescence background correction.

### Statistical Analysis

Statistical analysis was performed using GraphPad Prism 9 (GraphPad Software, Inc., La Jolla, CA, USA). Unless otherwise noted, the data are presented as Mean ± SEM. Comparisons of the two groups were performed by using a two-tailed unpaired Student’s *t* test. Comparisons of multiple groups at a single time point were performed by using a one-way analysis of variance (ANOVA). P values were presented as *P < 0.05; ** *P* < 0.01; *** *P* < 0.001; **** *P* < 0.0001.

## Supporting Information Available

Confocal imaging of OVA and OVA-DMPA antigen presentation on DCs, cell cytotoxicity of OVA and OVA-DMPA, BSA, and BSA-DMPA, Avidin and Avidin-DMPA, CD spectra of native and modified proteins, chemical structures of the ligands used in this work, physiochemical properties of native and modified proteins, cell penetration efficiency of native BSA and different ligands modified BSA, confocal imaging of cell penetration of native proteins BSA, mNeonGreen, Avidin, Cas13a, and their ligand modified proteins, absorbance spectra of avidin–biotin, and Cas13a RNase activity assay (PDF)
