# Supplementary material for: Efficient Direct Cytosolic Protein Delivery via Protein-Linker Co-engineering
Source: ACS Appl Mater Interfaces. 2025 Apr 30;17(19):27858–70. doi: 10.1021/acsami.5c02360 (PMC12086766; doi:10.1021/acsami.5c02360)
Supplement: Supplementary file 1 — am5c02360_si_001.pdf [file am5c02360_si_001.pdf]

Supporting Information

Efficient direct cytosolic protein delivery via protein-linker co-engineering

Lixia Wei[§†], Heyun Wang[§†], Melis Özkan[§†], Andrada-Ioana Damian-Buda[‡], Colleen N. Loynachan[§], Suiyang Liao[§†], Francesco Stellacci*[§†]

§ Institute of Materials Science and Engineering, École polytechnique fédérale de Lausanne, Lausanne, 1015, Switzerland
† Institute of Bioengineering, École polytechnique fédérale de Lausanne, Lausanne, 1015, Switzerland
‡ Institute of Biomaterials, Department Materials Science and Engineering, Friedrich-Alexander-Universität, Erlangen, 91054, Germany

*Email: francesco.stellacci@epfl.ch

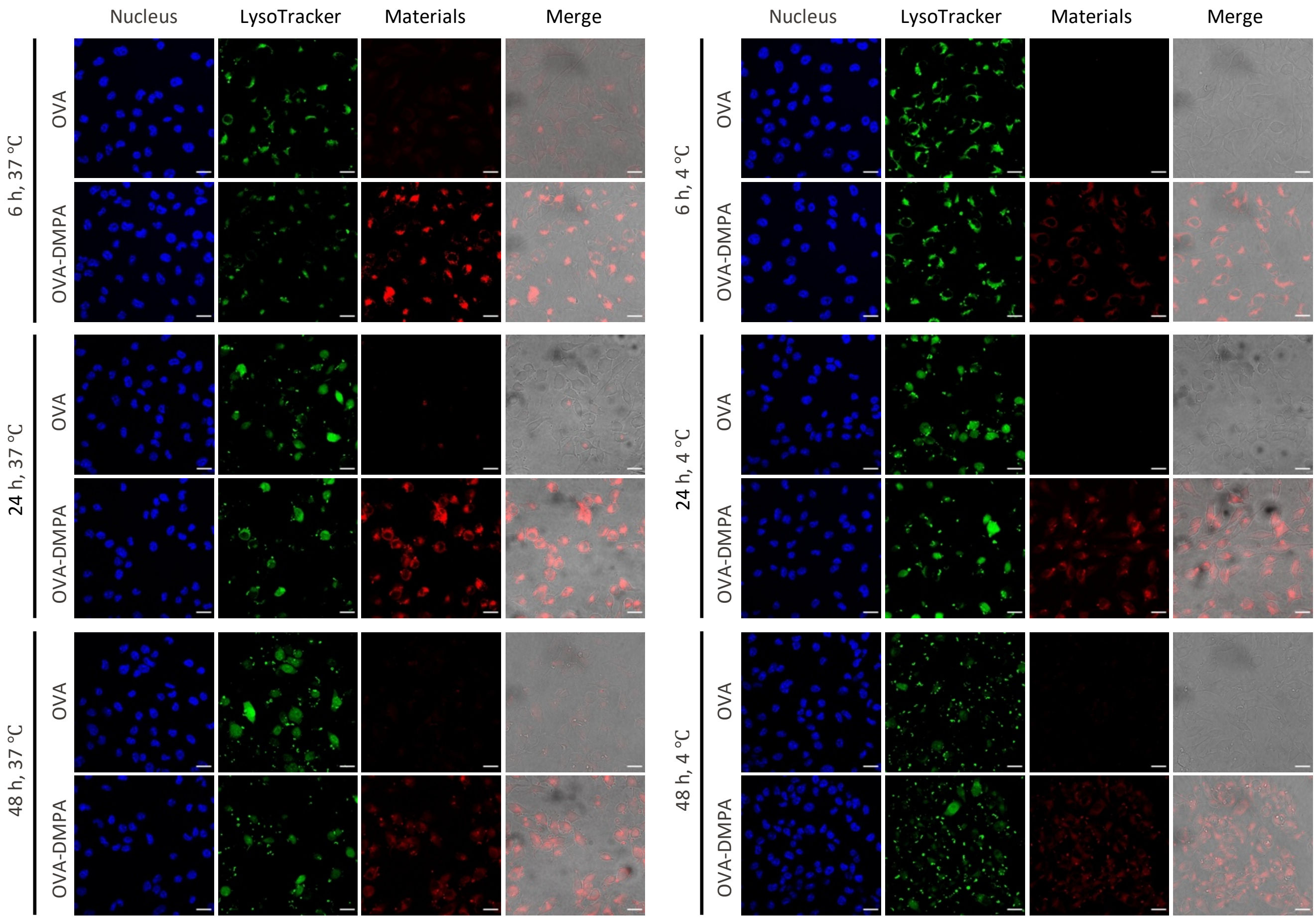

Figure S1. Confocal imaging visualization of native antigen OVA and modified OVA-DMPA antigen presentation on DCs at different time points (6h, 24h, and 48h) both at 37 °C and 4 °C. Scale bar: 20 μm.

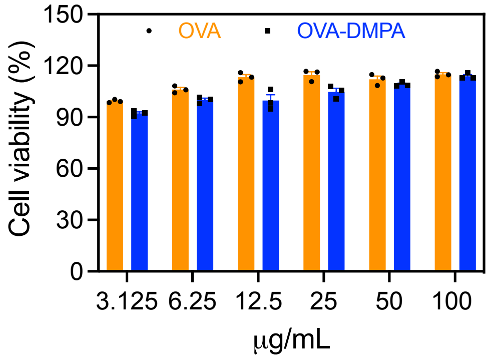

Figure S2. Cell cytotoxicity of native OVA and modified OVA-DMPA on DCs at different concentrations varying from 3.125 μg/mL to 100 μg/mL.

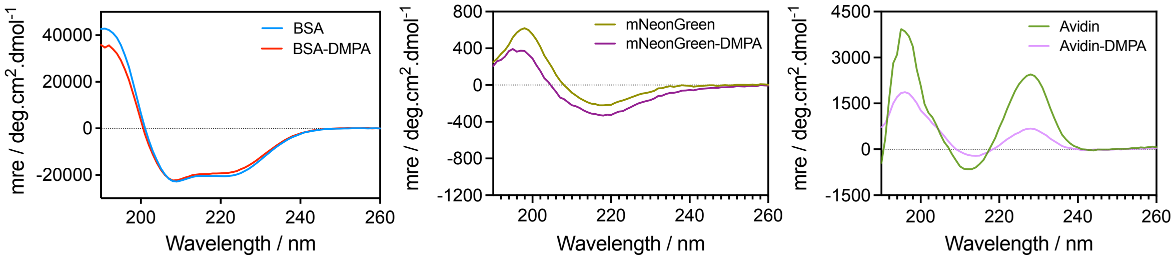

Figure S3. CD measurement of native BSA and modified BSA-DMPA, native mNeonGreen and modified mNeonGreen-DMPA, native Avidin, and modified Avidin-DMPA's secondary protein structures.

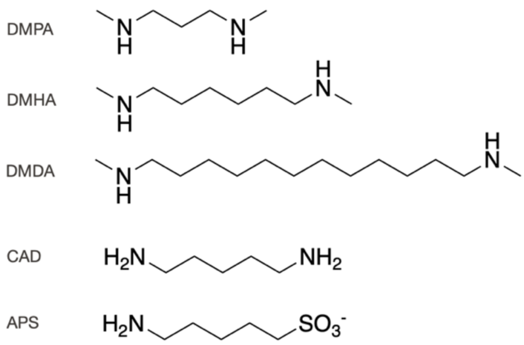

Scheme S1. Chemical structures of the ligands used in this work to study the ligand terminal group charge and hydrophobicity effects on protein cell penetration.

Table S1. Physicochemical Properties of native and ligand modified proteins.

Entry	Sample/ Characterization	DLS (nm)	Zeta potential (mV)	Mass Spectrum (MW)	Number of ligand / protein
1	BSA	6.1 ± 1.5	-7.3 ± 3.8	66346	
2	BSA-DMPA	8.2 ± 2.0	31.2 ± 4.5	72057	56
3	BSA-DMHA	7.4 ± 2.0	23.4 ± 6.0	71682	37
4	BSA-DMDA	7.7 ± 2.0	6.4 ± 14.6	71379	22
5	BSA-CAD	8.1 ± 0.5	29.7 ± 2.5	71290	47
6	BSA-APS	11.4 ± 1.7	-21.1 ± 1.7	72248	34

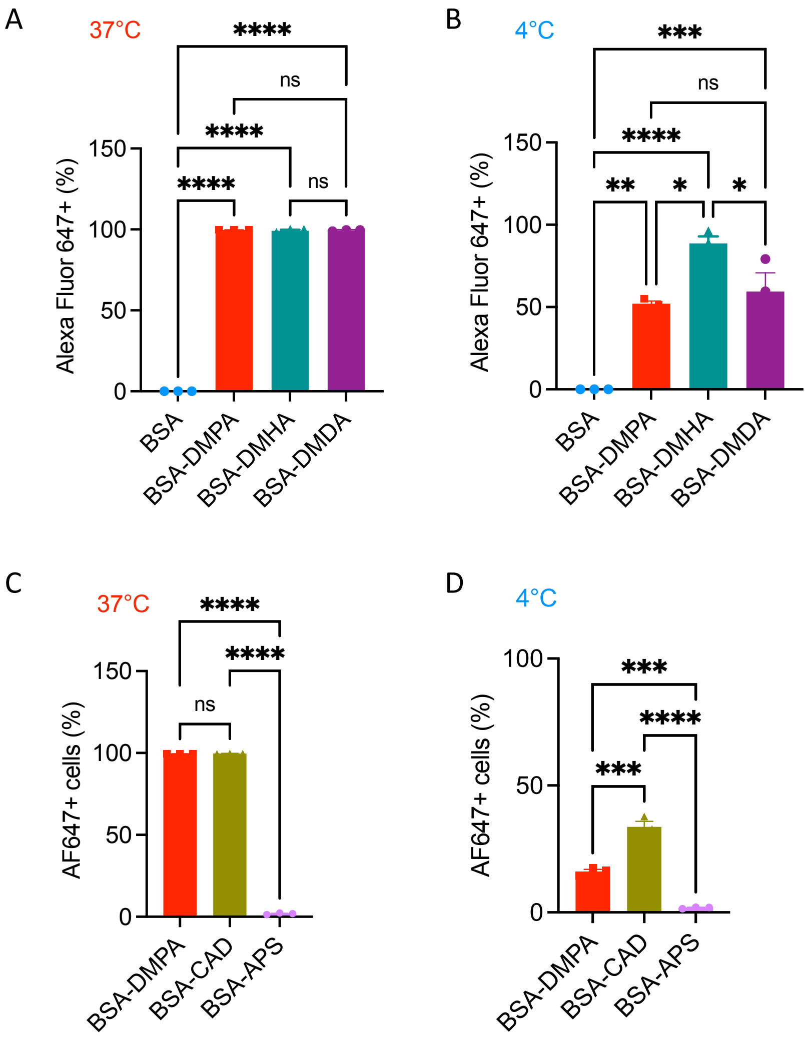

Figure S4. Cell penetration efficiency of ligand-modified BSA measured by flow cytometry on Hela cells. (A) Cell penetration of BSA modified with DMPA, DMHA, and DMDA ligands was measured at 37 °C. (B) Cell penetration of BSA modified with DMPA, DMHA, and DMDA ligands measured at 4 °C. (C) Cell penetration of BSA modified with CAD and APS ligands measured at 37 °C. (D) Cell penetration of BSA modified with CAD and APS ligands measured at 4 °C.

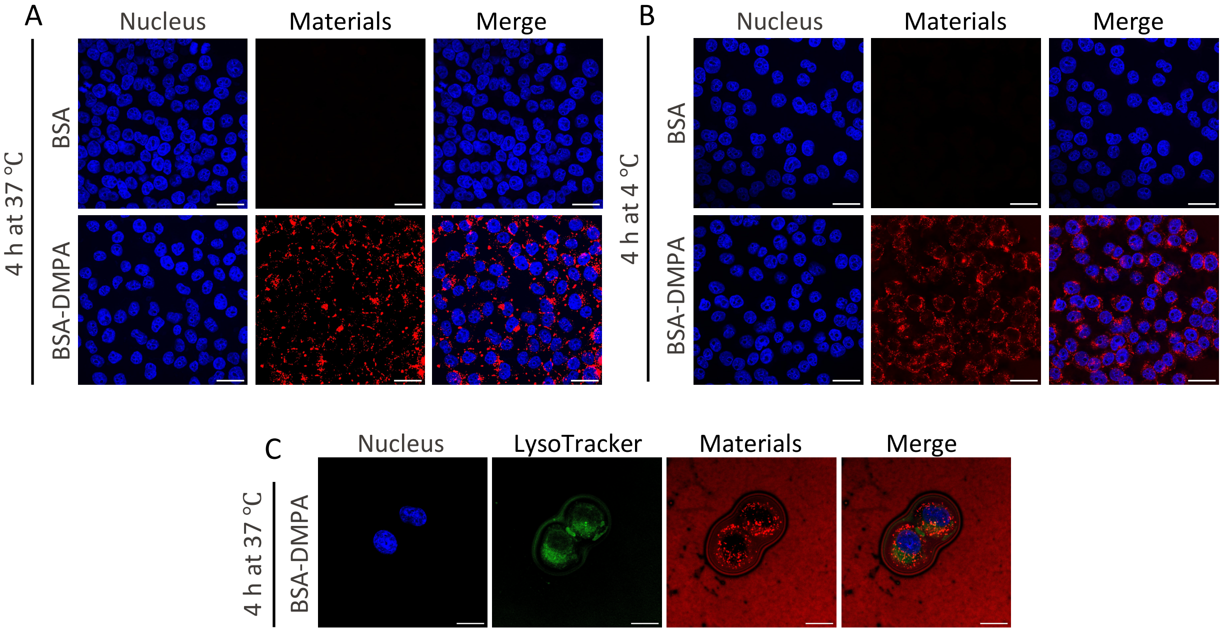

Figure S5. Confocal imaging visualization of Hela cell penetration of native BSA and modified BSA-DMPA. (A) at 37 °C, and (B) at 4 °C of BSA and BSA-DMPA for 4 hours. Scale bar: 30 μm. (C) Single-cell confocal images of modified BSA-DMPA penetrating Hela cells at 37 °C. Scale bar: 15 μm.

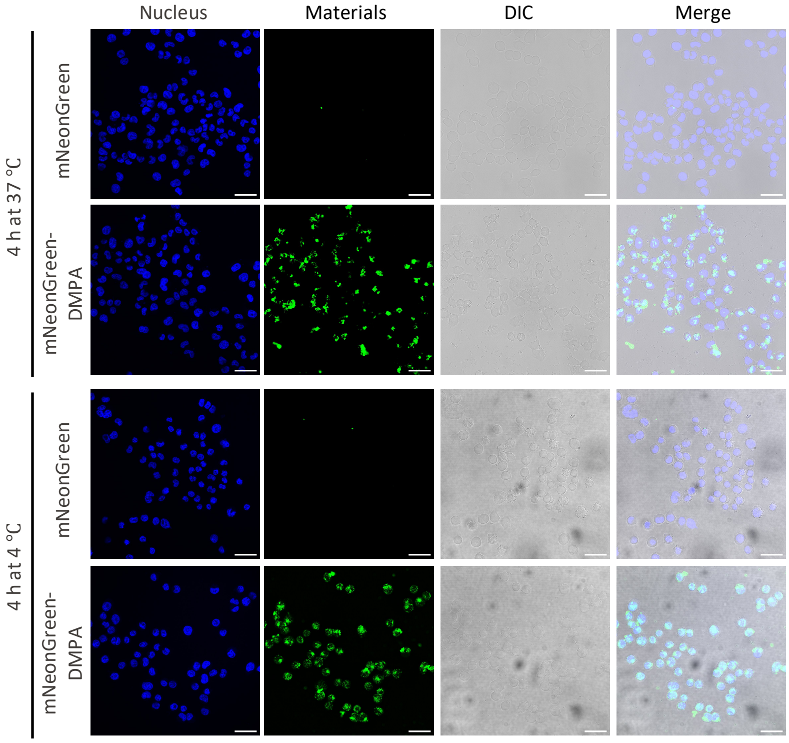

Figure S6. Confocal imaging visualization of Hela cell penetration of native mNeonGreen and modified mNeonGreen-DMPA both at 37 °C and 4 °C for 4 hours. Scale bar: 30 μm.

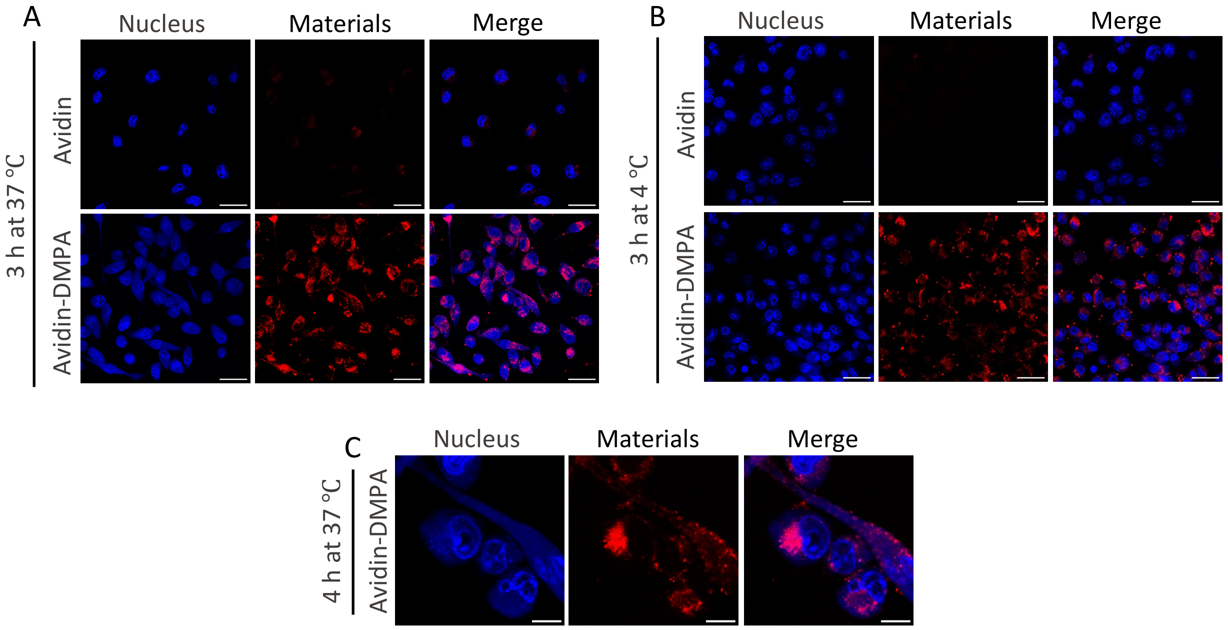

Figure S7. Confocal imaging visualization of Vero cell penetration of native Avidin and modified Avidin-DMPA. (A) at 37 °C, and (B) at 4 °C of Avidin and Avidin-DMPA for 3 hours. Scale bar: 30 µm. (C) Single-cell confocal images of modified Avidin-DMPA penetrating Vero cells at 37 °C. Scale bar: 8 µm.

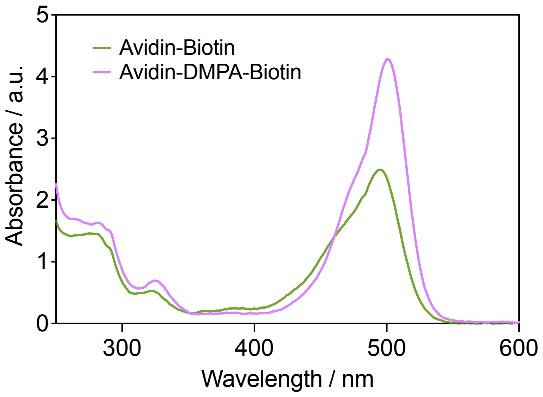

Figure S8. Absorbance at 490 nm of Avidin-Biotin Complex (ABC) for native Avidin and modified Avidin-DMPA.

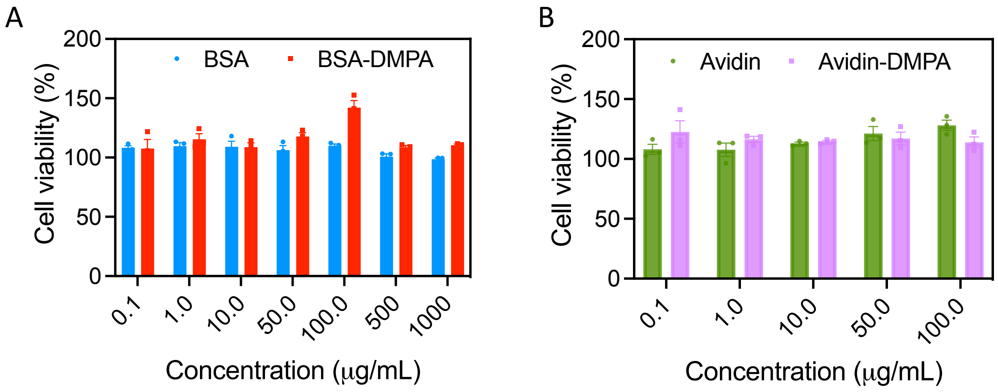

Figure S9. Cell cytotoxicity of native BSA and BSA-DMPA on Hela cells (A), native Avidin and modified Avidin-DMPA on Vero cells (B) at different concentrations.

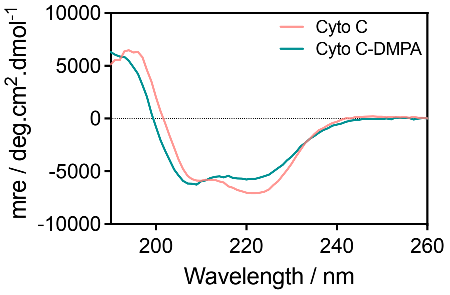

Figure S10. CD measurement of native Cyto C and modified Cyto C-DMPA's secondary protein structures.

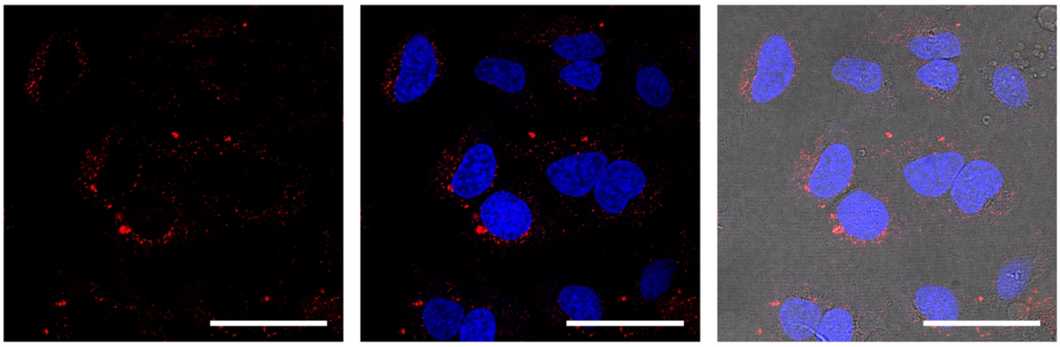

Figure S11. Representative confocal microscopy images of cell penetration of Cas13a-DMPA. Left: Cas13a-DMPA labeled with AlexaFluor 647; Middle: Cas13a-DMPA labeled with AlexaFluor 647 and DAPI; Right: Cas13a-DMPA labeled with AlexaFluor 647, DAPI and transmission images. Scale bar: 50 μm.

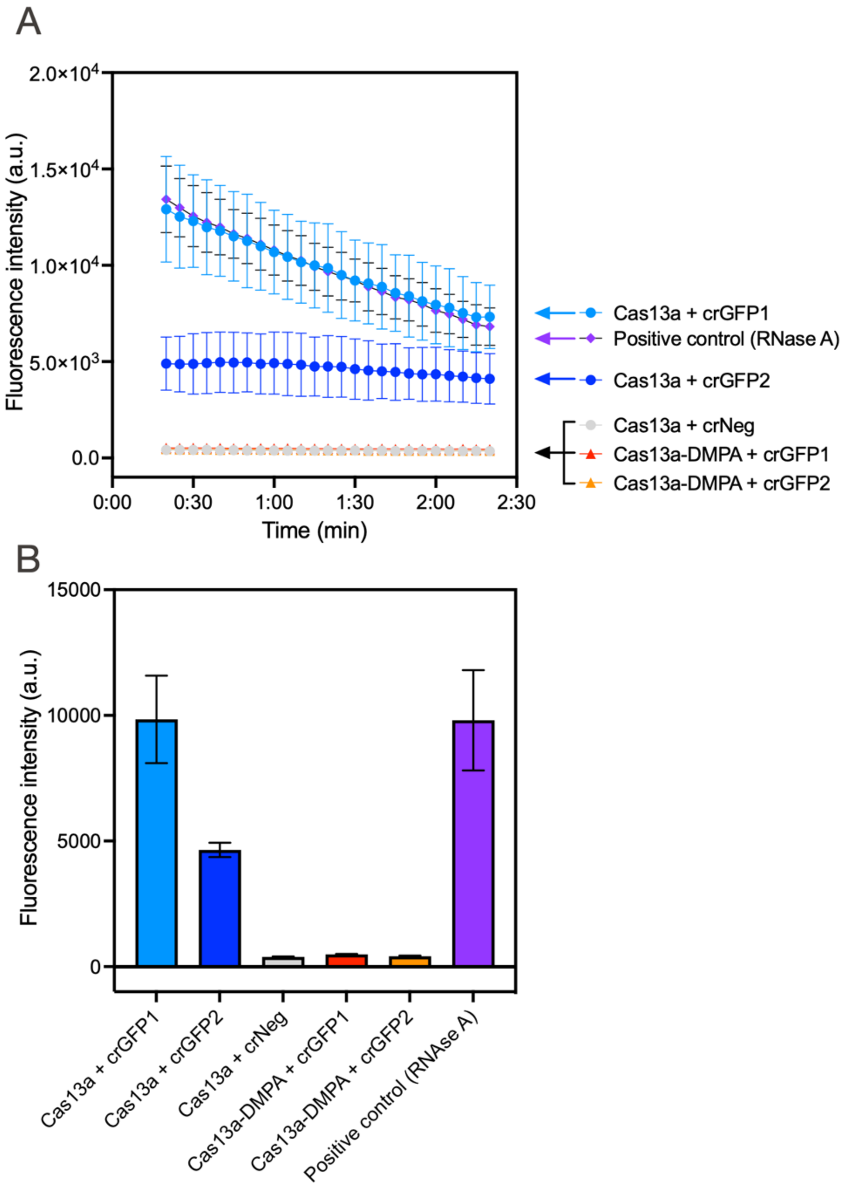

Figure S12. DMPA modification induces loss of RNase activity of Cas13a, assayed using the RNaseAlert v2 assay system (A) Kinetics curves of ex/em = 490/520 nm fluorescence intensity induced by the hydrolysis of self-quenching fluorescent probes (RNaseAlert v2). (B) Ex/em = 490/520 nm fluorescence intensity measured after 20 min incubation.
